# Does the inferior frontal sulcus play a functional role in deception? A neuronavigated theta-burst transcranial magnetic stimulation study

**DOI:** 10.3389/fnhum.2012.00284

**Published:** 2012-10-18

**Authors:** Bruno Verschuere, Teresa Schuhmann, Alexander T. Sack

**Affiliations:** ^1^Department of Clinical Psychology, University of AmsterdamAmsterdam, Netherlands; ^2^Experimental-Clinical and Health Psychology, Ghent UniversityGhent, Belgium; ^3^Clinical Psychology Science, Maastricht UniversityMaastricht, Netherlands; ^4^Cognitive Neuroscience, Maastricht UniversityMaastricht, Netherlands; ^5^Maastricht Brain Imaging CenterMaastricht, Netherlands

**Keywords:** deception, response inhibition, transcranial magnetic stimulation, theta-burst, inferior frontal sulcus

## Abstract

By definition, lying involves withholding the truth. Response inhibition may therefore be the cognitive function at the heart of deception. Neuroimaging research has shown that the same brain region that is activated during response inhibition tasks, namely the inferior frontal region, is also activated during deception paradigms. This led to the hypothesis that the inferior frontal region is the neural substrate critically involved in withholding the truth. In the present study, we critically examine the functional necessity of the inferior frontal region in withholding the truth during deception. We experimentally manipulated the neural activity level in right inferior frontal sulcus (IFS) by means of neuronavigated continuous theta-burst stimulation (cTBS). Individual structural magnetic resonance brain images (MRI) were used to allow precise stimulation in each participant. Twenty-six participants answered autobiographical questions truthfully or deceptively before and after sham and real cTBS. Deception was reliably associated with more errors, longer and more variable response times than truth telling. Despite the potential role of IFS in deception as suggested by neuroimaging data, the cTBS-induced disruption of right IFS did not affect response times or error rates, when compared to sham stimulation. The present findings do not support the hypothesis that the right IFS is critically involved in deception.

## Introduction

In recent years, deception researchers have focused upon the cognitive processes involved in deception (Vrij, 2008). Formulated broadly, the cognitive perspective on deception holds that deception is cognitively more demanding than truth telling. Deception often involves one or more of the following mental operations: the decision to lie, withholding the truth, fabrication of the lie, monitoring whether the receiver believes the lie and, if necessary, adjusting the fabricated story, and keeping the lying consistent. These operations make lying a cognitively demanding task. Evidence supports the cognitive perspective on deception. For example, lying participants were judged by observers to think harder than truthful participants, and participants subjectively reported more cognitive load when lying compared to truth telling (Vrij et al., 2006). Furthermore, compared to truth telling, lying is associated with more errors, increased and more variable response times (Spence et al., 2001; Johnson et al., 2005; Verschuere et al., 2011). Recently, several studies used brain imaging techniques such as fMRI (Spence et al., 2001; Langleben et al., 2002; Ganis et al., 2003; Kozel et al., 2005; Phan et al., 2005; Monteleone et al., 2006; Abe et al., 2008), PET (Abe et al., 2006), and fNIRS (Tian et al., 2009) to identify which brain regions are associated with deception. Common across these studies is the greater activation in the prefrontal cortex during lying compared to truth telling (Christ et al., 2009), thereby supporting the idea that deception requires greater executive control than truth telling.

Since deception by definition involves withholding the truth, response inhibition may be crucial for deception. Indeed, liars may or may not overtly express a deceitful answer, but they definitely need to refrain from telling the truth. Response inhibition can be defined as the cognitive function that allows one to intentionally inhibit a dominant, automatic or prepotent response (Miyake et al., 2000). The truth, then, is regarded as the dominant response that needs to be actively inhibited in order to lie (Spence et al., 2008a). Noteworthy from the perspective of the association between response inhibition and deception, is the observation that the same brain regions are critically involved in response inhibition and in deception. Examining the neural correlates of response inhibition, imaging studies have examined brain activity during tasks that require active suppression of a dominant response such as the Go/No-Go task and the Stop-signal task. The Go/No-Go task requires a speeded response to frequently presented Go trials (e.g., the letter Q), but inhibition of responding to the rarely presented No-Go trials (e.g., the letter O). In the Stop-signal, responding to the go task (e.g., press left for circle and right for square) has to be inhibited when an auditory signal is presented. A particular region in the prefrontal cortex, the right inferior frontal region, is consistently and most strongly activated during such tasks (Garavan et al., 1999; Konishi et al., 1999; Aron et al., 2004; Brass et al., 2005). In 18 patients with right frontal lobe damage, it was found that the greater the damage to the inferior frontal gyrus (IFG), the worse response inhibition performance in the Stop-signal task (Aron et al., 2003). Further support for the functional necessity of the IFG in response inhibition comes from recent work using repetitive transcranial magnetic stimulation (rTMS). rTMS is a non-invasive brain stimulation technique that allows to induce a transient and reversible “virtual lesion” in healthy conscious volunteers. rTMS to the IFG, but not to mid frontal or parietal regions, impaired response inhibition capacity in healthy volunteers (Chambers et al., 2006). As the inferior frontal region is also consistently activated in deception paradigms (Spence et al., 2001; Kozel et al., 2005; Phan et al., 2005; Gamer et al., 2007; Christ et al., 2009), it may be this region that is crucial for inhibiting the truth during deception (Spence et al., 2004).

In sum, brain imaging studies suggest that the inferior frontal region may exert a functional role in withholding the truth during deception. However, since imaging studies are in essence correlation studies, they do not allow conclusions with regard to the functional necessity of brain regions. In order to investigate the functional necessity of this region for deception, one would need to experimentally manipulate its activity level and investigate the impact on deception (Sack, 2006; Luber et al., 2009). Here, we present the first study that used rTMS to unravel the functional relevance of the inferior frontal region for deception. Following recent imaging data (Brass et al., 2005), we focused upon the right inferior frontal sulcus (IFS). We collected structural images of the brain using magnetic resonance imaging (MRI). These individual anatomical brain images were used as a basis for a frameless stereotaxic TMS neuronavigation system, allowing us to precisely map and target the IFS with TMS in each individual participant. Furthermore, we used an innovative TMS protocol, continuous theta-burst rTMS (cTBS), that requires a much shorter stimulation time yet leads to more robust inhibitory after-effects than conventional rTMS protocols (Huang et al., 2005; Thut and Pascual-Leone, 2010). Disruption of the right IFS using cTBS impairs stopping performance in a stop-signal task (Verbruggen et al., 2010). This MRI-guided cTBS neuronavigation approach was used here to transiently disrupt neural processing in the right IFG to examine whether it is causally related to deception.

## Materials and methods

### Participants

Thirty-one participants were paid €15/h for participation. All participants had normal or corrected-to-normal vision and had no history of neurological or psychiatric disorders. They received medical approval for participation and gave their written informed consent after being introduced to the procedure. The study was approved by the local Medical Ethical Commission, and written informed consent was obtained from all participants.

Due to experimenter error, data from three participants were lost. Furthermore, the data from one participant for whom rTMS was stopped after a startle response were excluded. Finally, data from one participant were excluded because of an excessive error percentage (18%; >2.5 *SD*s from the *M*).

The final sample consisted of 26 participants (15 women, 11 men; *M*_age_ = 26.11 years, *SD* = 7.53; 96% right-handed). Participants were tested in their preferred language (19 Dutch, 6 English, and 1 French).

### Procedure

Participants were tested in three separate sessions. In session 1, we obtained anatomical brain measurements of all participants using MRI. In session 2, participants were informed about the experiment and rTMS, filled in the autobiographical questionnaire, and performed the deception test a first time. Next, the active motor threshold (AMT) for each participant was determined. We then used frameless stereotaxy for MRI-guided TMS neuronavigation to the previously defined target region, and applied either a cTBS protocol that has shown to inhibit the stimulated areas for up to 1 h following the TBS itself (Huang et al., 2005; Thut and Pascual-Leone, 2010), or sham TBS using a placebo TMS coil. The second deception test followed immediately after the rTMS/sham stimulation. The procedure was identical for session 3, except that stimulation type differed and that motor threshold was not determined again. Real rTMS stimulation was on day 1 for 15 participants and on day 2 for 11 participants.

This study design and methodological approach enabled us to first define the target brain area based on the individual anatomical data and to subsequently neuronavigate the TMS coil to the anatomically defined stimulation site in each participant. The MRI-guided TMS neuronavigation was monitored online throughout the whole stimulation time, allowing for a precise determination of the actual stimulation site also during stimulation.

#### Deception paradigm: the sheffield lie test

The Sheffield lie test is a “differentiation of deception” paradigm (Furedy et al., 1988) that was developed by Spence and colleagues from Sheffield University (Spence et al., 2001, 2008a,b), and has been successfully replicated by our group (Verschuere et al., 2011) and others (Fullam et al., 2009). Participants first completed a questionnaire that listed 72 specific behaviors (e.g., “Bought a newspaper”), and were asked to indicate whether or not they had performed those actions that day. Half of these questions came from the study by Spence et al. (2001) the remaining half were developed for the present study. Trials in the Sheffield lie test consisted of statements from the autobiographical questionnaire presented for 5 s. Participants answer the statements with a right-hand *Yes* or *No* response. The *Yes* and *No* reminder labels remained on the screen throughout the test. Crucially, their color varied after every six trials. One color (e.g., yellow) indicated the participant to answer truthfully, whereas the other (e.g., blue) was the signal to lie, with colors counterbalanced across participants. Meaning of the colors was assigned in the instructions, and checked in a practice phase with statements for which ground truth was known (e.g., “Are you in France?”). The test consisted of 72 trials, with each of 36 statements appearing once with blue and once with yellow reminder labels. After a 5 min break, participants took the deception test again, this time without practice at the beginning. One set of 36 questions was used in the first test, and one set in the second test, with sets counterbalanced across participants. These sets were tested beforehand to result in a deception effect of similar magnitude. Statements were presented by a PC using Inquisit 3.0 software (Inquisit, 2009).

#### MRI measurements

A high-resolution anatomical image was obtained from each participant in a 3-T magnetic resonance scanner (Siemens Allegra MR Tomograph; Siemens AG, Erlangen, Germany) at the Faculty of Psychology and Neuroscience, Maastricht University, The Netherlands. The data set was acquired with the help of a T1-weighted structural scan with an isotropic resolution of 1 mm using a modified driven equilibrium Fourier transform (MDEFT) sequence with optimized contrast for GM and WM and imaging parameters.

#### Cortical-surface reconstruction

Data were analyzed using the BrainVoyager QX 2.0 software package (BrainInnovation, Maastricht, The Netherlands). The high-resolution anatomical recordings were used for surface reconstruction of the right hemisphere of each participant (Kriegeskorte and Goebel, 2001). The surface reconstruction was performed in order to recover the exact spatial structure of the cortical sheet and to improve the visualization of the anatomical gyrification. The white-gray-matter boundary was segmented with a region growing method preceded by inhomogeneity correction of signal intensity across space. The borders of the two resulting segmented subvolumes were tessellated to produce a surface reconstruction of the right hemisphere.

#### TMS apparatus and stimulation parameters

Biphasic TMS pulses were applied using the MagProX100 stimulator (Medtronic Functional Diagnostics A/S, Sklovunde, Denmark) and a figure-of-8 coil (MC-B70, inner radius 10 mm, and outer radius 50 mm) for real stimulation. The maximum output of this coil and stimulator combination is approximately 1.9 Tesla and 150 A/μS. A specific figure-of-8 placebo coil (MC-P-B70) was also employed in order to reproduce the same acoustic stimulation as the active coil while not inducing the magnetic field (sham stimulation). The coil was manually held tangentially to the skull with the coil handle oriented perpendicular to the posterior part of the IFS using the online visualization function of the BrainVoyager TMS Neuronavigator. Following Huang et al. (2005), continuous theta-burst TMS was applied at 80% AMT. A detailed description of this rTMS paradigm can be found in Huang et al. (2005). In brief, in TBS protocols, short bursts of 50 Hz rTMS are repeated at a rate in the theta range (5 Hz) as a continuous (cTBS) or intermittent (iTBS) train (Huang et al., 2005; Di Lazzaro et al., 2008). Depending on the train intervals, TBS can either have longer-lasting facilitatory or inhibitatory after effects. The after effects of TBS were found to be significantly longer-lasting compared to conventional rTMS (Huang et al., 2005) with shorter stimulation time and lower stimulation intensity needed. These factors could allow more comfortable stimulation conditions, especially when TBS is used as a therapeutical intervention over a long period of time (Cardenas-Morales et al., 2010). It has been suggested that cTBS decreases the effectiveness of synaptic connections that are recruited in circuits involved in both short interval intracortical inhibition (SICI) and intracortical facilitation (ICF) (Huang et al.). Some side effects were noted with this stimulation, most notably muscle twitches at the eye, cheek and mouth.

#### TMS localization

**Figure 1:**
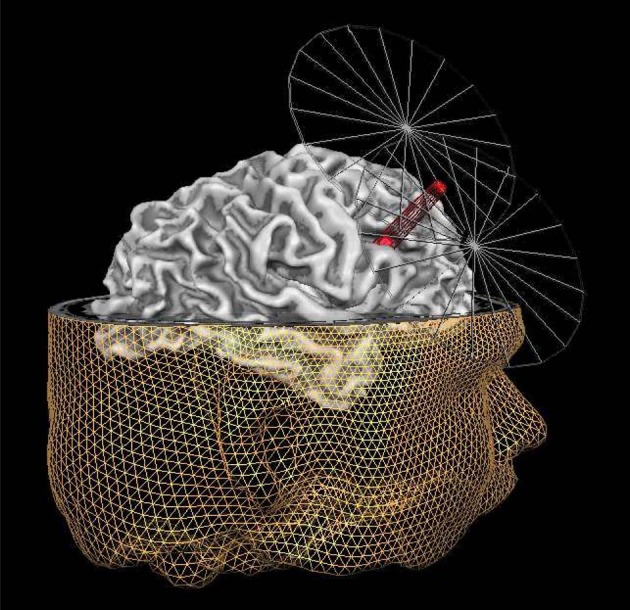
**Graphic representation of the MRI neuronavigated C-TBS at the right IFS**. The inferior frontal sulcus (IFS) target point (red dot under the beam of the coil) for TMS, shown on the reconstruction of the right hemisphere of one exemplary participant. The target point is placed on the posterior part of the right IFS, in particular the area just anterior to the section of the precentral sulcus and the inferior frontal sulcus. In addition to the reconstruction of the right hemisphere of this participant, also the reconstruction of the head is displayed together with a simplified visualization of the coil. The tip of the red beam from the TMS figure-8 coil indicates the site of the maximal stimulation.

IFS corresponds to area 44 in Brodmann's cytoarchitectonic map (Brodmann, 1909). Based upon anatomical landmarks, we targeted the posterior part of the right IFS. Specifically, we targeted the area just anterior to the section of the precentral sulcus and the IFS. The stimulation site was localized using frameless stereotaxy (Brain Voyager TMS neuronavigation; Sack et al., 2006) for both real and sham stimulation. Using such a TMS neuronavigation system enabled us to account for inter-individual differences in anatomical brain structures while stimulating (see Figure 1).

#### TMS procedure

Individual AMTs were determined as the intensity at which the stimulation of the left motor cortex with single-pulse TMS resulted reliably in a visible movement of the first dorsal interosseous (FDI) muscle. The AMT of the participants ranged from 21 to 45% of maximum stimulator output [*M* = 30.27% (47 A/μS), *SD* = 5.24]. The mean stimulation intensity was set at 80% of the AMT and therefore resulted in 24.19% (38 A/μS) of maximum stimulator output (range 17–36%, *SD* = 4.97). Throughout the stimulation time, participants were wearing earplugs to protect their ears from the clicking sound and to minimize the interference of sounds during the task.

## Results

Separate 2 × 2 × 2 ANOVAs with stimulation (rTMS vs. sham), session (pre vs. post), and deception (lie vs. truth) as the within-subjects factors were conducted on error percentage (%), and on mean (RTs) and variability (SD RTs) of correct response times.

### Errors

**Figure 2:**
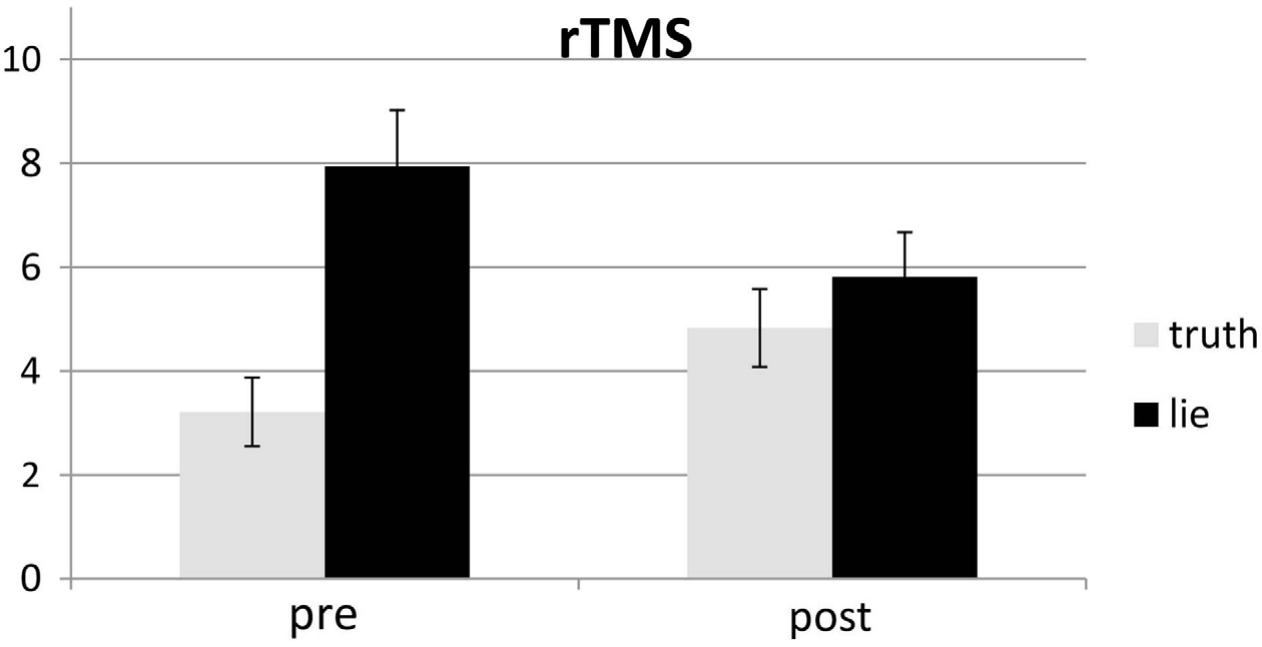
**Mean error (in %; ± one SE) for lie and truth trials, pre and post real rTMS**.

**Figure 3:**
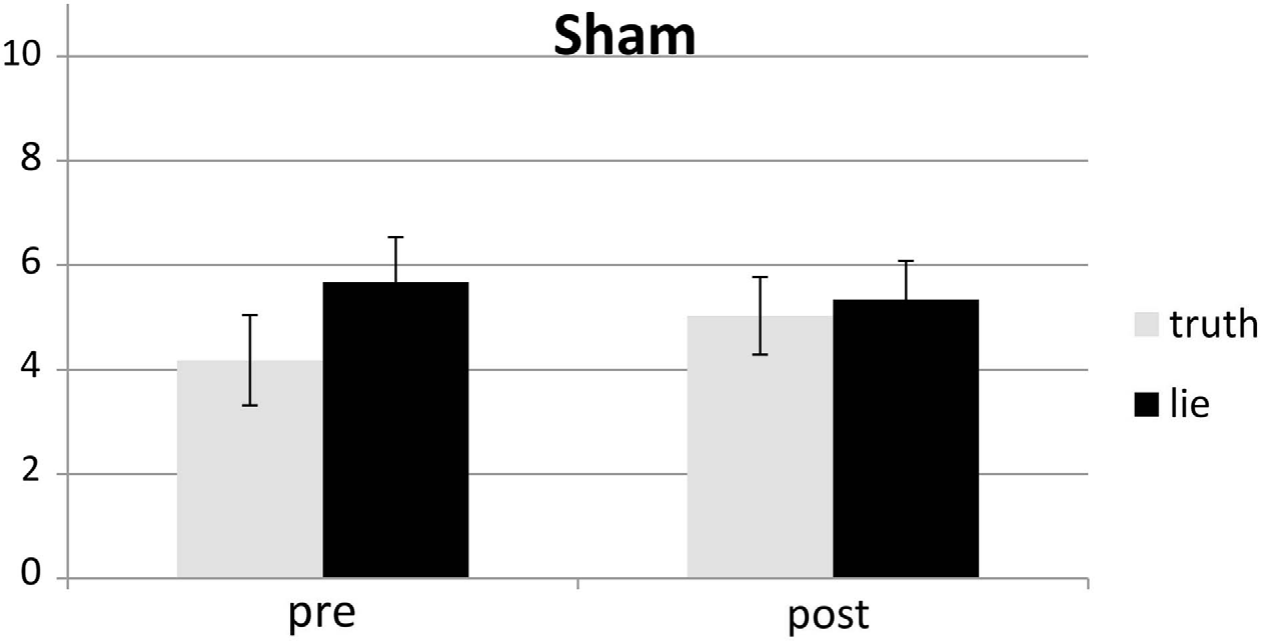
**Mean error (in %; ± one SE) for lie and truth trials, pre and post sham**.

Responses that did not match with the autobiographical questionnaire were considered behavioral errors. The only reliable effect was a main effect of deception, *F*_(1, 25)_ = 10.22, *p* < 0.01, with lying resulting in more errors than truth telling, see Figures 2, 3. Two other effects just failed short of reaching significance: Session × Deception, *F*_(1, 25)_ = 4.15, *p* = 0.05, indicating that the lie vs. truth difference was somewhat greater at baseline than at test; and Stimulation × Deception, *F*_(1, 25)_ = 3.06, *p* = 0.09, indicating that the lie vs. truth difference was somewhat greater in the rTMS session than in the sham session. Other *F*'s < 1.

### RTs

**Figure 4:**
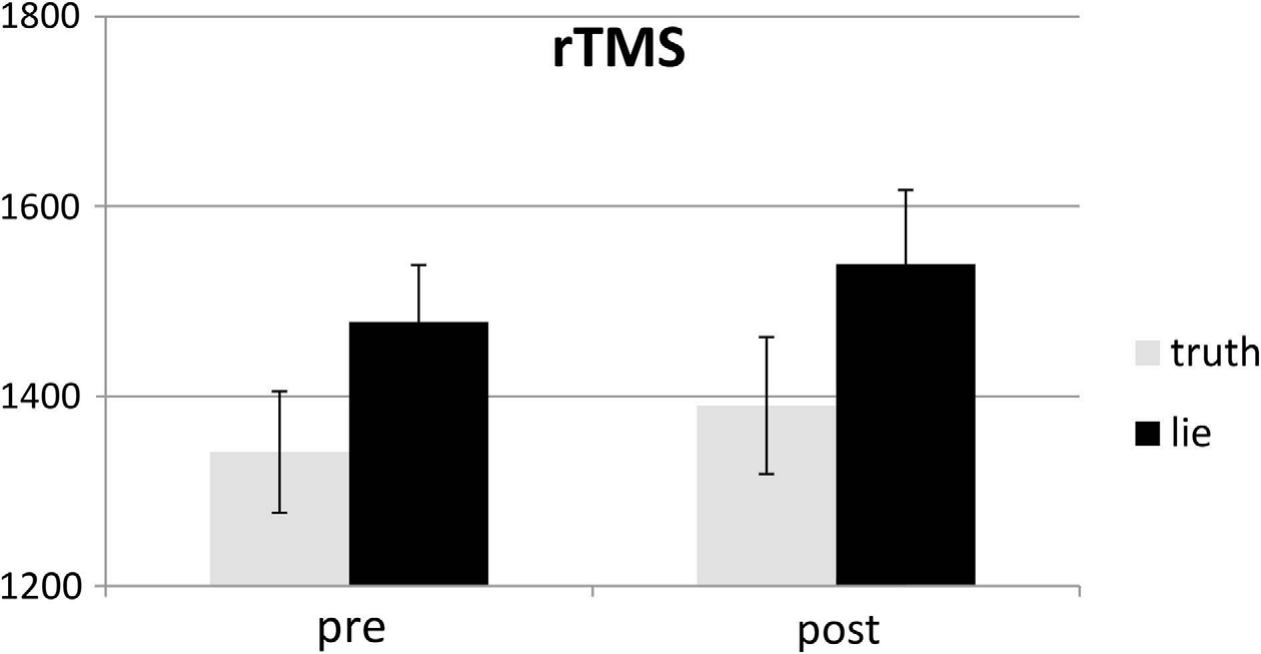
**Mean RTs (ms; ± one SE) for lie and truth trials, pre and post real rTMS**.

**Figure 5:**
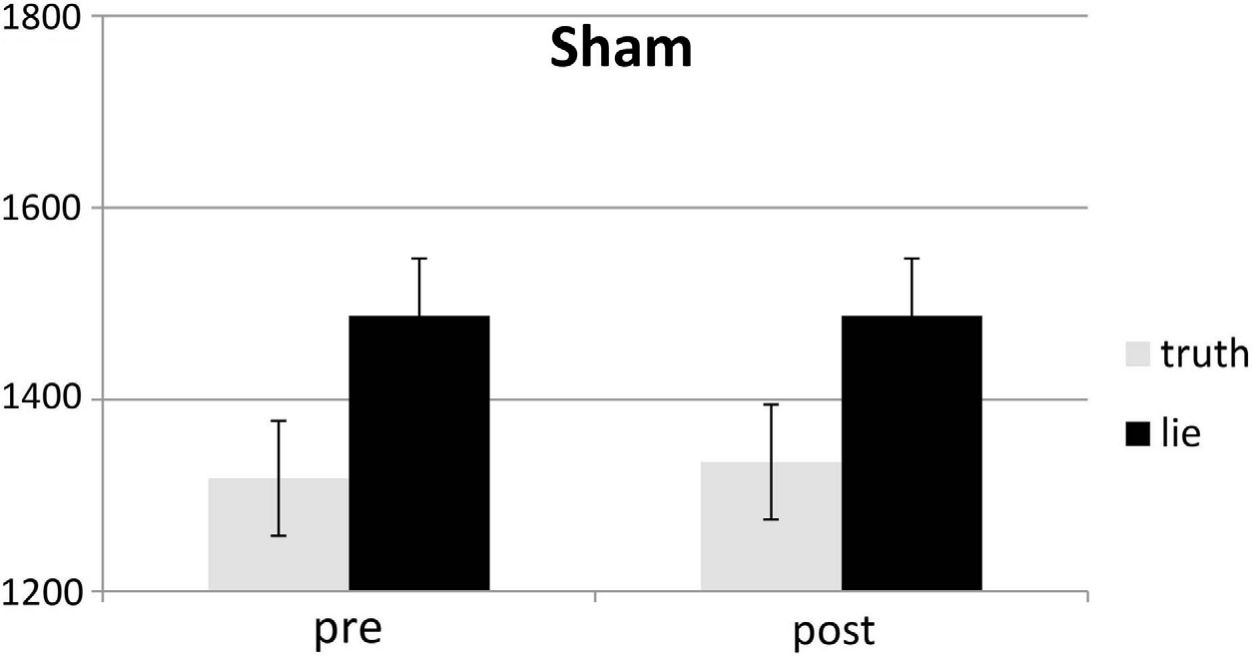
**Mean RTs (ms; ± one SE) for lie and truth trials, pre and post sham**.

Behavioral errors were excluded from the RT analyses, as where RTs that deviated more than 2.5 *SD*s from the individual conditional mean (Ratcliff, 1993). There was only a main effect of deception, with participants being slower when lying than when telling the truth, *F*_(1, 25)_ = 43.19, *p* < 0.001, see Figures 4, 5. Other *F*'s < 1.5.

### SD RTs

**Figure 6:**
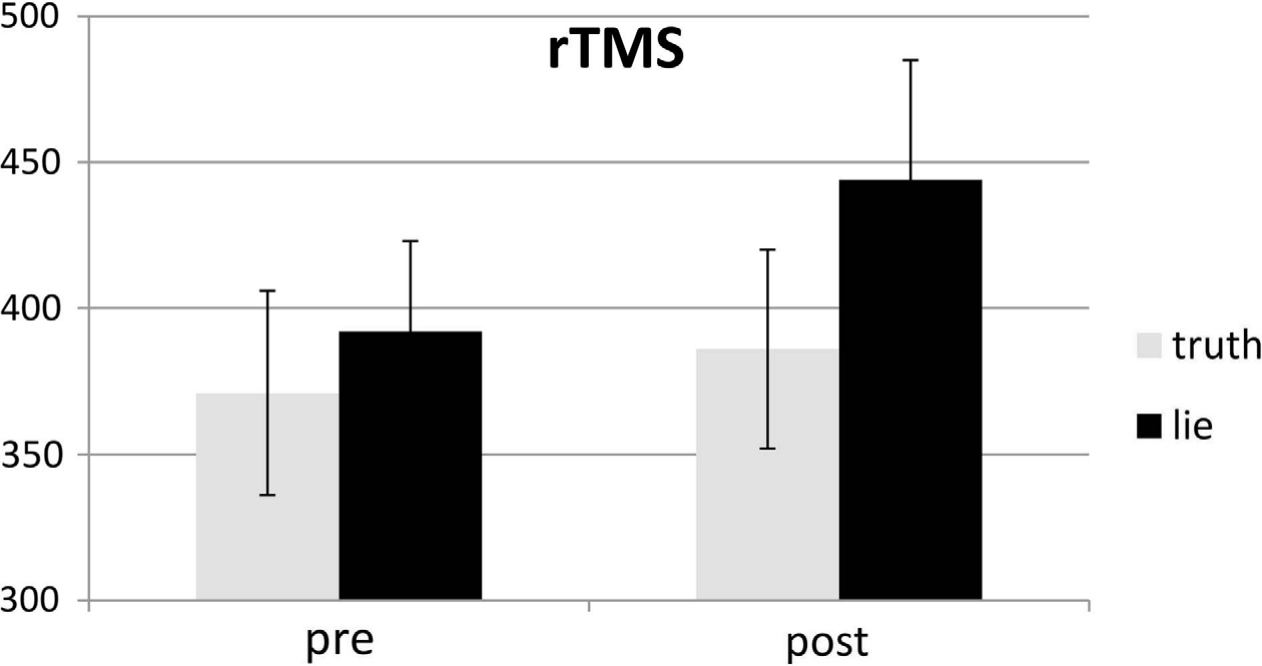
**Mean SDs of RTs (ms; ± one SE) for lie and truth trials, pre and post real rTMS**.

**Figure 7:**
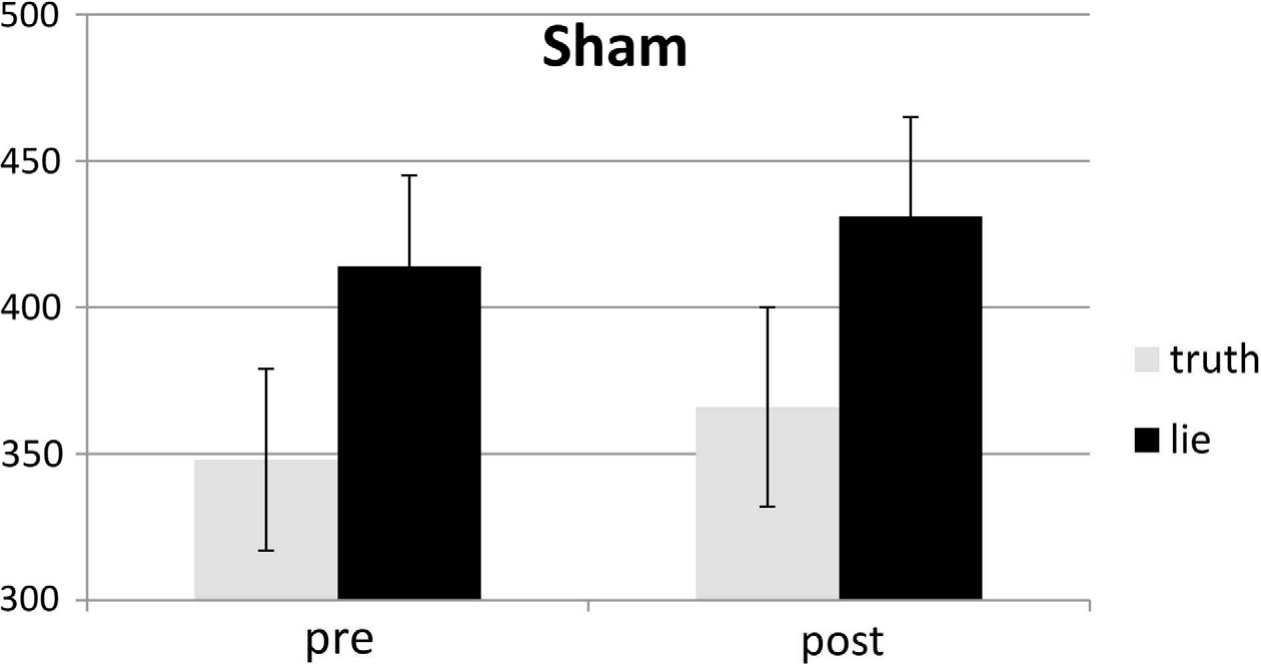
**Mean SDs of RTs (ms; ± one SE) for lie and truth trials, pre and post sham**.

SD RTs of the RTs included in the RT analyses were analyzed. There was only a main effect of deception, with participants being slower when lying than when telling the truth, *F*_(1, 25)_ = 13.31, *p* < 0.01, see Figures 6, 7. Other *F*'s < 2.2.

## Discussion

Since deception by cognitive definition involves withholding the truth, response inhibition may be the cognitive function at the heart of deception. The behavioral data in the present study indeed showed that lying comes with a “cost,” as lying was reliably associated with more errors and greater and more variable response times compared to truth telling, thereby replicating previous findings obtained with the Sheffield lie test (Spence et al., 2001, 2008a; Fullam et al., 2009; Verschuere et al., 2011) as well as with other deception paradigms (e.g., Sartori et al., 2008; Verschuere et al., 2009). A prominent cognitive neurobiological account of deception holds that this cost can be related to the active inhibition of the dominant truth response (Spence et al., 2008a), and that this response inhibition of the truth is regulated mainly in right inferior frontal cortex (Spence et al., 2001, 2004; Kozel et al., 2005; Phan et al., 2005; Gamer et al., 2007; Christ et al., 2009). Being in essence correlation studies, imaging studies do not allow conclusions with regard to the functional necessity of brain regions. Here, we used rTMS to unravel the functional relevance of the right inferior frontal cortex for deception, expecting that a cTBS-induced disruption of right IFS would affect behavioral responding on the lying trials. However, real cTBS over right IFS had no effect on deception as compared to sham stimulation in the current study.

Our present findings failed to refute the null hypothesis, leaving us with the question whether the data can be meaningfully interpreted or not (De Graaf and Sack, 2011). To the extent that methodological aspects can explain our negative findings, interpretation is hazardous. Under certain methodological conditions, however, negative TMS findings provide a meaningful answer to the question that cannot be answered by imaging techniques: Is the specific brain region functionally relevant for the task or not? After all, TMS is an entirely different method than brain imaging, going beyond the correlation approach, and allowing to examine whether a region identified in imaging work is functionally relevant for the task or may be a non-functional by-product. Three important aspects need consideration to make meaningful interpretation of negative TMS findings (De Graaf and Sack, 2011): the localization argument (perhaps the coil was not positioned properly and the targeted brain region X was therefore not stimulated), the neural efficacy argument (did the expected neural effects occur?), and the power argument (maybe a non-significant TMS effect requires more participants). The *power argument* is not easily refuted, but is unlikely to explain our negative findings given the lack of statistical trends, and the use of a within-subjects design that seems sufficiently powered (*n* = 26) compared to previous research (Huang et al., 2005; Chambers et al., 2006; Verbruggen et al., 2010). With regard to the *localization argument*, the current study used individual MRI data to neuronavigate the coil to a specific individually-defined target point within IFS (see the “Materials and Methods” section). While we cannot rule out that individual fMRI data may have resulted in a slightly different TMS target site and potentially different results, we can conclude that stimulating the anatomical region within IFS shown here (Figure 1) does not affect deception. With regard to the *neural efficiency argument*, the question can be raised whether the stimulation produced the intended change in cortical excitability. Unlike for the motor system, no direct and easily measurable assessment for the local cortical excitability level of right IFS is available, unless cTBS is directly combined with EEG or fMRI during stimulation. It has been shown that there is a considerable inter-individual variance in the cortical after effects of rTMS (Maeda et al., 2000) with some participants showing an increase in cortical excitability while others showing a respective decrease in cortical excitability, even when being stimulated with the same rTMS protocol. Moreover, it has been shown that the same rTMS protocol can induce opposite neural after effects (excitatory vs. inhibitory) when applied over different cortical target sites (Paus et al., 1997). Future research will benefit from direct concurrent neurophysiologic measurements to examine the direction of the change in cortical excitability induced by the rTMS/ transcranial direct current stimulation (tDCS) intervention. Furthermore, future studies should also include other control sites, and not only make use of sham stimulation as a control, since participants might be able to detect the difference between real and sham stimulation.

Whereas we cannot easily dismiss all methodological arguments relating to power, localization, and neural efficiency our negative findings may be meaningfully interpreted given that our study was based on a clear a priori hypothesis directly derived from the imaging literature, and conducted using state-of-the art TMS methodology—including (1) the employment of individual structural brain imaging data to select and target the right IFS in each individual participant, (2) a paradigm that reliably elicits stronger inferior frontal activation for lying compared to truth telling (Spence et al., 2001; Christ et al., 2009; Fullam et al., 2009), (3) a reasonably powered design (within-subjects; *n* = 26), and (4) a stimulation protocol (cTBS) that has been shown to produce immediate, profound and lasting effects on cognitive functioning generally and on inhibition specifically (Huang et al., 2005; Thut and Pascual-Leone, 2010; Verbruggen et al., 2010). As such our finding that our inhibitory protocol (cTBS) over the right IFS identified by individual MRI (see target site in Figure 1) did not have behavioral effects on deception as measured within the Sheffield lie test contains much more information than a “pure” null result and is informative for the scientific community. The present study rejoins a handful of neuromodulation studies on deception. Unfortunately, the results of these studies are mixed and inconsistent. In the present study, we failed to find an effect of cTBS to the rIFC on deception. Previous studies have used related technique: tDCS or rTMS, both of which can be used to either increase or decrease neural excitability. Priori et al. (2008) unexpectedly found that anodal (excitatory) tDCS of the DLPFC *hampered* lying, with no effect of cathodal (inhibitory) stimulation. Karim et al. (2010), however, failed to find an effect of anodal tDCS to the anterior PFC. Rather, they found that cathodal tDCS to the same region *facilitated* lying. Rather than hampering lying as observed by Priori et al. (2008), Mameli et al. (2010) found that anodal tDCS of the DLPFC *facilitated* lying. Finally, Karton and Bachmann (2011) found that inhibiting the left DLPFC using low frequency rTMS makes people less truthful, whereas inhibiting the right DLPFC makes them more truthful. The small sample size (*n* = 8), and the lack of a baseline assessment are noteworthy shortcomings of this latter study. Taken together, these studies point to a functional role of the DLPFC in deception, yet also underscore that its exact role remains unclear. Interestingly, rTMS studies of deception have received great media attention, headings “Magnets, the ultimate truth serum”, “Scientists can make you lie using magnets,” and “Magnetic pulses to the brain make it impossible to lie.” Our findings together with our review of previous rTMS studies of deception show these headline are misleading. Clearly, we are far from using this technology in applied setting, because we do not know exactly whether and how neuromodulation will affect lying ability. Still, neuromodulation is a powerful and promising technique that may help to reveal the neural underpinnings of deception. We hope that the present report provides an impetus to further investigate the functional necessity of brain regions associated with deception (Christ et al., 2009) using rTMS/tDCS.

### Conflict of interest statement

The authors declare that the research was conducted in the absence of any commercial or financial relationships that could be construed as a potential conflict of interest.
